# Prognostic value of near-infrared spectroscopy regional oxygen saturation and cerebrovascular reactivity index in acute traumatic neural injury: a CAnadian High-Resolution Traumatic Brain Injury (CAHR-TBI) Cohort Study

**DOI:** 10.1186/s13054-024-04859-6

**Published:** 2024-03-14

**Authors:** Alwyn Gomez, Logan Froese, Donald Griesdale, Eric P. Thelin, Rahul Raj, Levi van Iperenburg, Jeanette Tas, Marcel Aries, Kevin Y. Stein, Clare Gallagher, Francis Bernard, Andreas H. Kramer, Frederick A. Zeiler

**Affiliations:** 1 Department of Human Anatomy and Cell Science, Rady Faculty of Health Sciences, University of Manitoba, Winnipeg, Canada; 2 Section of Neurosurgery, Department of Surgery, Rady Faculty of Health Sciences, University of Manitoba, Winnipeg, MB Canada; 3 Department of Biomedical Engineering, Price Faculty of Engineering, University of Manitoba, Winnipeg, MB Canada; 4 Department of Anesthesiology, Pharmacology and Therapeutics, University of British Columbia, Vancouver, BC Canada; 5 Department of Neurology, Karolinska University Hospital, Stockholm, Sweden; 6 Department of Clinical Neuroscience, Karolinska Institutet, Stockholm, Sweden; 7 Department of Neurosurgery, University of Helsinki and Helsinki University Hospital, Helsinki, Finland; 8 Department of Intensive Care, Maastricht University Medical Center+, Maastricht, The Netherlands; 9 School of Mental Health and Neurosciences, University Maastricht, Maastricht, The Netherlands; 10 Section of Neurosurgery, Department of Clinical Neurosciences, Hotchkiss Brain Institute, University of Calgary, Calgary, AB Canada; 11 Department of Clinical Neurosciences, University of Calgary, Calgary, AB Canada; 12 Hotchkiss Brain Institute, University of Calgary, Calgary, AB Canada; 13 Section of Critical Care, Department of Medicine, University of Montreal, Montreal, QC Canada; 14 Department of Critical Care Medicine, University of Calgary, Calgary, AB Canada; 15 Centre on Aging, University of Manitoba, Winnipeg, Canada; 16 Division of Anaesthesia, Department of Medicine, Addenbrooke’s Hospital, University of Cambridge, Cambridge, UK

**Keywords:** Cerebrovascular reactivity, Multimodal monitoring, Near-infrared spectroscopy, Traumatic brain injury

## Abstract

**Background:**

Near-infrared spectroscopy regional cerebral oxygen saturation (rSO_2_) has gained interest as a raw parameter and as a basis for measuring cerebrovascular reactivity (CVR) due to its noninvasive nature and high spatial resolution. However, the prognostic utility of these parameters has not yet been determined. This study aimed to identify threshold values of rSO_2_ and rSO_2_-based CVR at which outcomes worsened following traumatic brain injury (TBI).

**Methods:**

A retrospective multi-institutional cohort study was performed. The cohort included TBI patients treated in four adult intensive care units (ICU). The cerebral oxygen indices, COx (using rSO_2_ and cerebral perfusion pressure) as well as COx_a (using rSO_2_ and arterial blood pressure) were calculated for each patient. Grand mean thresholds along with exposure-based thresholds were determined utilizing sequential chi-squared analysis and univariate logistic regression, respectively.

**Results:**

In the cohort of 129 patients, there was no identifiable threshold for raw rSO_2_ at which outcomes were found to worsen. For both COx and COx_a, an optimal grand mean threshold value of 0.2 was identified for both survival and favorable outcomes, while percent time above − 0.05 was uniformly found to have the best discriminative value.

**Conclusions:**

In this multi-institutional cohort study, raw rSO_2_was found to contain no significant prognostic information. However, rSO_2_-based indices of CVR, COx and COx_a, were found to have a uniform grand mean threshold of 0.2 and exposure-based threshold of − 0.05, above which clinical outcomes markedly worsened. This study lays the groundwork to transition to less invasive means of continuously measuring CVR.

**Supplementary Information:**

The online version contains supplementary material available at 10.1186/s13054-024-04859-6.

## Background

Contemporary critical care management of moderate-to-severe traumatic brain injury (TBI) remains focused on guideline-based universal intracranial pressure (ICP) and cerebral perfusion pressure (CPP) targets [1, 2]. Despite various iterations, outcomes in these critically ill patients have failed to improve over the past decades [3]. As a result, attention is shifting toward a multimodal monitoring approach in hopes of better understanding individual patient-level cerebral pathophysiologic states [4]. However, prior to driving management strategies, a thorough understanding of outcome associations of these various parameters must be developed.

One such modality of interest is near-infrared spectroscopy (NIRS). Near-infrared light has the ability to penetrate past the scalp and skull down to the cerebral parenchyma where it is scattered or absorbed by various chromophores depending on the wavelength of light emitted [5, 6]. In spatially resolved NIRS devices, light scattered from oxyhemoglobin and deoxyhemoglobin within the cerebral microvasculature can be detected, isolated, and utilized to calculate parameters such as regional cerebral oxygen saturation (rSO_2_).

While advantageous due to its noninvasive nature and relatively high spatial resolution, the association of NIRS parameters and outcomes following TBI remains unclear [7]. There is a substantive body of the literature supporting an association between NIRS parameters and cerebral blood flow (CBF) but threshold values at which outcomes worsen have not been identified [8].

On the other hand, dysfunctional cerebral autoregulation/cerebrovascular reactivity (CVR) as a contributor to secondary injury following TBI has been greatly explored [9–12]. This has been largely facilitated by the development of continuous measures of CVR, the most prominent of which being the pressure reactivity index (PRx). PRx leverages ICP monitoring, typically indicated in the setting of moderate and severe TBI, as a surrogate for pulsatile cerebral blood volume (CBV) and arterial blood pressure (ABP) as a surrogate for driving pressure. Values closer to − 1 are indicative of a vasoactive state (i.e., intact CVR), and values closer to + 1 are indicative of a vasopassive state (i.e., disrupted CVR) [13].

A large body of evidence has validated the relationship between dysfunctional CVR, as measured by PRx, and poor functional outcomes following moderate-to-severe TBI [14–22]. Further, specific thresholds of PRx have been identified at which outcomes tend to worsen and mortality increases following TBI [22–24]. This has ultimately led to studies trying to therapeutically maintain PRx below critical thresholds [25–29].

For all its strengths, PRx does have its inherent limitations. Foremost, its reliance on invasive ICP monitoring limits its application to settings where ICP monitoring is indicated. Thus, the use of PRx beyond the acute phase, or in milder TBIs, is limited. Additionally, ICP can only act as a global surrogate for CBV and therefore the spatial resolution of PRx is minimal with essentially only whole brain assessment being feasible. As a result, alternative means of evaluating local CVR continuously at the bedside have become of interest.

While NIRS-based parameters have had mixed evidence surrounding their utility in TBI care, leveraging these parameters to calculate metrics of CVR holds promise. In fact, NIRS-based indices of CVR are the only other continuous indices outside of ICP-based indices that have been shown to identify the lower limit of autoregulation in large animal models [30–32]. However, threshold values, at which clinical outcomes worsen, have not been identified.

This study explores the outcome associations of rSO_2_, both as a raw NIRS parameter and as a surrogate for CBV in NIRS-based indices of CVR, with outcomes. It leverages the largest multicenter high-frequency physiologic data set with NIRS concurrently recorded with ICP and ABP in moderate-to-severe TBI, from the CAnadian High-Resolution TBI (CAHR-TBI) Research Collaborative. The primary aim of this study was to identify the associations between these parameters and clinical outcomes following moderate-to-severe TBI. The secondary aim of this study was to identify threshold values for these parameters at which clinical outcomes worsen.

## Methods

### Study design

A retrospective multicenter cohort study was performed leveraging prospectively collected high-resolution physiologic data and clinical outcome data from the CAHR-TBI Research Collaborative [33]. The CAHR-TBI Research Collaborative is a multi-institutional collaboration pooling high-resolution physiologic data recordings from TBI patients admitted for moderate-to-severe TBI. The associated database contains physiologic data recordings dating back to 2011 and has prospective data collection ongoing. Local ethics approval for all aspects of data collection and anonymous data transfer between centers was obtained from the individual local research ethics boards: University of Manitoba Biomedical Research Ethics Board (BREB, H2017:181, H2017:188, H2020:118, B2023:001), University of Calgary Conjoint Health Research Ethics Board (CHREB, H20-03759), University of British Columbia Clinical Research Ethics Board (CREB, REB20-0482) and University of Maastricht Medical Ethics Committee (16-4-243).

### Patient population

The CAHR-TBI database includes high-resolution physiologic data collected from moderate-to-severe TBI patients, defined as admission Glasgow Coma Scale (GCS) of less than 13, admitted to adult intensive care units (ICU) at participating institutions [33]. For the purposes of this retrospective study, data were collected at four university-affiliated hospitals: Foothills Medical Centre (University of Calgary), Health Sciences Centre Winnipeg (University of Manitoba), Maastricht University Medical Center (University of Maastricht), and Vancouver General Hospital (University of British Columbia). Patient demographics (age and biologic sex), admission injury severity characteristics (GCS and pupil reactivity), and imaging characteristics (Marshall CT score) were all collected. Finally, 6-month outcome data were also collected using the Extended Glasgow Outcome Scale (GOSE). Data regarding withdrawal of care were not available; however, as per local treatment guideline at each center, if care was decided to be withdrawn after the appropriate discussion with family/proxy, monitoring was discontinued. As such, physiologic data were only obtained during periods of active treatment. Given the exploratory and retrospective nature of this study, sample size calculations were not able to be performed and all datasets that met inclusion criteria were utilized for analysis.

Subjects from the CAHR-TBI database were included in this study if they had ICP, ABP, and rSO_2_ monitoring. Recording was initiated within 24 h of time from injury. All patients received standard care based on published guidelines [1]. This included placement of ICP monitors, when indicated, and therapeutic treatment of ICP values greater than 20–22mmHg. A CPP target of greater than 60mmHg was also utilized. Notably, elevated CPP (> 70mmHg) was not routinely treated as per local practice. While NIRS-based rSO_2_ was collected on all included patients, it was not routinely utilized to guide clinical care. Finally, individual patient care was not guided by continuous CVR metrics.

### High-resolution physiologic data collection

ICP was monitored utilizing an intraparenchymal probe (Codman ICP MicroSensor, Codman & Shurtleff Inc., Raynham, MA, USA; or NEUROVENT-P-TEMP, Raumedic Inc., Hambrecht, Germany; or Comino ICP monitor, Natus Medical Inc., Middleton, WI, USA) placed in the frontal lobe or with an external ventricular drain (EVD; Medtronic, Minneapolis, MN). No correction was made for monitor drift. ABP was obtained using a radial arterial line connected to a pressure transducer (Baxter Healthcare Corp. CardioVascular Group, Irvine, CA, USA) zeroed at the level of the tragus. rSO_2_ was collected using NIRS regional cerebral oximetry of both the left and right frontal lobes (INVOS 5100C or 7100, Covidien-Medtronic, Minneapolis, MN), where possible.

Data streams were recorded in digital high-frequency time series (≥ 100Hz for ABP and ICP, oversampled at 1Hz for rSO_2_, while signal generation was 0.2Hz) using analogue-to-digital signal converters (Data Translations, DT9804 or DT9826) when required. Digitized data were linked and stored in time series using Intensive Care Monitoring (ICM +) software (Cambridge Enterprise Ltd, Cambridge, UK).

### Physiologic data cleaning and processing

All high-resolution physiologic data were cleared manually by qualified personnel utilizing ICM + software. This was done without knowledge of study objectives or patient demographic data to minimize bias. This included removal of periods when the EVD was open to drainage, identified by complete loss of ICP waveform.

Following artifact clearing, data were down-sampled utilizing a 10-s non-overlapping moving average filter to eliminate high-frequency fluctuations in these parameters and focus on vasogenic slow-wave fluctuations associated with cerebral vasomotion [17, 34]. Next, CVR indices were derived utilizing 300 s window Pearson correlations between surrogates of CBV (ICP or rSO_2_) and surrogates of driving pressure (ABP or CPP), continuously updating every minute. Utilizing this method, PRx was derived (correlation between ICP and ABP) along with two variations of the cerebral oxygen index: COx (correlation between rSO_2_ and CPP) and COx_a (correlation between rSO_2_ and ABP). ICP, ABP, rSO_2_, PRx, COx, and COx_a were exported as minute-by-minute comma-separated values (*.csv*) files for each patient. No interpolation of missing data was performed as it was not required for subsequent analysis.

To provide the best quality data and simplify analysis, a single side/channel of rSO_2_ was utilized for analysis and was selected to avoid interference from extravascular blood (scalp, epidural, subdural, or intraparenchymal hematomas/contusions) as based on radiographic imaging. If a subject had evidence of bilateral extravascular blood, they were excluded from analysis. If no imaging data were available, the side with the longest duration of rSO_2_ recording was selected to maximize data while maintaining a single NIRS channel. It was felt that averaging channels would introduce too much variability in the data during periods when one channel would drop out, which is a frequent occurrence in the setting of trauma.

### Physiologic data analysis and statistical methods

R statistical software (Version 4.3.1, R Foundation for Statistical Computing, Vienna, Austria) was utilized for all data analysis leveraging the *tidyverse* and *fmsb* packages. OpenBLAS (Version 0.3.20, Institute of Software, Chinese Academy of Sciences, Beijing, China) was utilized for the Basic Linear Algebra Subprograms (BLAS) and the Linear Algebra Package (LAPACK) to improve multithreaded computational performance.

Patients were categorized based on survival at 6-months and based on favorable (GOSE 5–8) or unfavorable (GOSE 1–4) clinical outcome at 6-months. Mean values of ICP, rSO_2_, PRx, COx, and COx_a were computed for each patient over the course of their entire recording period in ICU. Physiologic and demographic data were summarized for the entire cohort based on median values and interquartile ranges (IQR), or number of subjects, where appropriate.

Demographic and mean physiologic data were compared between alive/dead and favorable/unfavorable groupings utilizing Mann–Whitney U and chi-squared testing. Next, univariate logistic regression analysis was performed to determine the prognostic utility of averages of the physiologic parameters in isolation. This was followed by multivariable logistic regression analysis to characterize the prognostic value of the various physiologic parameters in and above a base model comprising of known clinical and radiographic prognostic indicators from standard prognostic models (age, admission GCS, admission pupil exam, and Marshall CT score) [35]. For the univariate and multivariable regression analyses, area under the receiver operating characteristic curve (AUC), Akaike information criterion (AIC), and Nagelkerke R^2^ values were also determined to help compare the various models. Given the exploratory nature of this study, alpha was set to 0.05 without correction for multiple comparisons.

Following analyses previously reported in the multimodal monitoring TBI literature, derivation of threshold values of rSO_2_, COx, and COx_a, at which survival and favorable outcomes worsened, was attempted [23, 24, 36]. Sequential 2 × 2 tables were generated based on patient outcome (either alive/dead or favorable/unfavorable) and whether the average physiologic parameter was above or below threshold. Sequential threshold values were utilized incrementing them by 0.05 for COx and COx_a and by 5% for rSO_2_. For all three physiologic parameters, Pearson’s chi-squared values were calculated for the 2 × 2 tables generated at each threshold. For each parameter examined, the threshold value that produced the largest statistically significant chi-squared value was deemed to have the best discriminative value.

While average values over a recording period may be useful prognostically, identification of thresholds for which percent time above/below is most discriminative may help better inform bedside management. To identify these exposure-based thresholds, the percentage time below rSO_2_ threshold and above COx and COx_a threshold was calculated for each subject. Sequential threshold values were again utilized incrementing them by 0.05 for COx and COx_a and by 5% for rSO_2_. A series of univariate logistic regressions were performed for both survival and favorable outcome (Survival ~ Percent time over COx Threshold, Survival ~ Percent time over COx_a Threshold, Survival ~ Percent time below rSO_2_ Threshold, Favorable Outcome ~ Percent time over COx Threshold, Favorable Outcome ~ Percent time over COx_a Threshold, Favorable Outcome ~ Percent time below rSO_2_ Threshold) with the various threshold values. To determine the ideal threshold for each parameter, the area under the receiver operator characteristic curve (AUC) was calculated for each model and compared. The threshold values that produced the highest AUC were deemed to have the best discriminative value.

## Results

### Cohort demographics

**Fig. 1:**
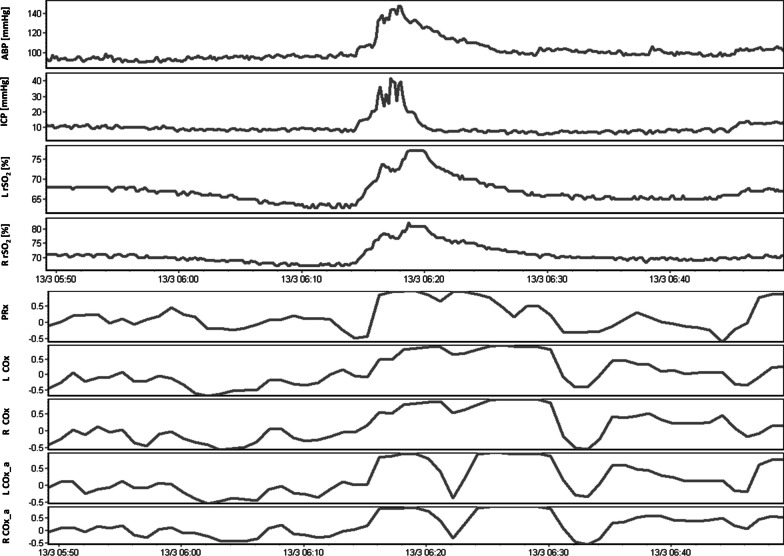
A sample of the 10-s-by-10-s recorded parameters arterial blood press (ABP), intracranial pressure (ICP), and left and right regional cerebral oxygen (L rSO_2_ and R rSO_2_) can be seen in the first four series. The minute-by-minute derived cerebrovascular reactivity indices (PRx—pressure reactivity index, L Cox—left cerebral oxygen index based on cerebral perfusion pressure, R COx—right cerebral oxygen index based on cerebral perfusion pressure, L COx_a—left cerebral oxygen index based on ABP, R COx_a—right cerebral oxygen index based on ABP) can be seen in the lower five series. Note that all five CVR indices trend well with each other

In total, 129 subjects from the CAHR-TBI database were included in the study with admission to hospital between November 2016 and December 2022. This included 18 patients from Foothills Medical Centre (University of Calgary), 87 patients from Health Sciences Centre Winnipeg (University of Manitoba), 22 patients from Maastricht University Medical Center (University of Maastricht), and 2 patients from Vancouver General Hospital (University of British Columbia). In total 751,060 min of unique physiologic recording was collected with a median recording duration of 4578 min (IQR: 2346 to 8275 min) per subject. A 1-h sample of the 10-s-by-10-s moving averaged physiologic data and minute-by-minute derived CVR indices can be seen in Fig. 1. Radiographic data to identify extravascular blood for the purposes of selecting rSO2 channel side were available in 87 subjects. The right rSO_2_ channel was selected in 102 subjects, while the left channel was selected in 27 subjects. Demographic and physiologic data for the cohort are summarized in Table 1.

**Table 1:** Patient demographics and cerebral physiology

Demographic parameter	Median or number of patients *N* = 129
Age (IQR)	41 (28–57)
Male, sex (%)	102 (79.1)
Admission GCS (IQR)	6 (4–8)
Admission pupils	
Bilaterally reactive (%)	91 (70.5)
Unilaterally reactive (%)	20 (15.5)
Bilaterally unreactive (%)	18 (14.0)
N/A (%)	0 (0.0)
Marshall CT classification	
I (%)	2 (1.6)
II (%)	31 (24.0)
III (%)	30 (23.3)
IV (%)	19 (14.7)
V (%)	46 (35.7)
VI (%)	0 (0.0)
N/A, *n* (%)	1 (0.8)
Follow-up 6-month GOSE	
1 (%)	51 (39.5)
2 (%)	1 (0.8)
3 (%)	7 (5.4)
4 (%)	2 (1.6)
5 (%)	12 (9.3)
6 (%)	14 (10.9)
7 (%)	28 (21.7)
8 (%)	11 (8.5)
N/A, *n* (%)	3 (2.3)
Subject average ICP in mmHg (IQR)	10.0 (6.9–13.6)
Subject average CPP in mmHg (IQR)	73.5 (69.1–79.6)
Subject average rSO_2_ in % (IQR)	69.7 (64.1–75.1)
Subject average PRx (IQR)	0.14 (0.02–0.25)
Subject average COx (IQR)	0.02 (-0.04–0.08)
Subject average COx_a (IQR)	0.04 (0.01–0.11)
Duration of ICP recording (days)	2.8 (1.1–4.9)
Duration of CPP recording (days)	2.7 (1.0–4.7)
Duration of rSO_2_ recording (days)	2.5 (1.1–4.9)
Subject average PaCO_2_ (mmHg)*	37 (34–40)
Subject average PaO_2_ (mmHg)*	111 (89–140)

*COx* Cerebral Oxygen Index Based on Cerebral Perfusion Pressure; *COx_a* Cerebral Oxygen Index Based on Arterial Blood Pressure; *CPP* Cerebral Perfusion Pressure; *CT* Computed Tomography; *GCS* Glasgow Coma Scale; *GOSE* Extended Glasgow Outcome Scale; *ICP* Intracranial Pressure; *IQR* Interquartile Range; *PaCO*_*2*_ Partial Pressure of Arterial Carbon Dioxide; *PaO*_*2*_ Partial Pressure of Arterial Oxygen; *PRx* Pressure Reactivity Index; and *rSO*_*2*_ Regional Cerebral Oxygen Saturation

*Data only available from 87 patients

The results of the Mann–Whitney-U testing between alive/dead and favorable/unfavorable groups can be seen in Table 2. Age was found to be statistically higher in those who died and those who had an unfavorable outcome. GCS on admission was found to be lower in those who had an unfavorable outcome at follow up. This trend was seen when examining survival; however, this did not reach statistical significance. All measures of CVR (PRx, COx, Cox_a) were found to be statistically higher in those who died. While PRx and COx trended toward being higher in those with an unfavorable outcome than those with a favorable one, this did not reach statistical significance. Finally, of note, rSO_2_ was no different across survival or clinical outcome groups.

**Table 2:** Comparison of demographic and physiologic parameters across alive/dead and favorable/unfavorable groups

Variable	Favorable/unfavorable			Alive/dead		
	Favorable median (IQR) or number (%) *N* = 67	Unfavorable median (IQR) or number (%) *N* = 62	*p* value	Alive median (IQR) or number (%) *N* = 78	Dead median (IQR) or number (%) *N* = 51	*p* value
Age	38.5 (27.0–50.0)	49.0 (32.0–66.0)	**0.0040**	38.0 (25.3–38.3)	57.0 (35.3–66.8)	**< 0.0010**
Male sex	51 (77.3)	50 (80.6)	0.64	61 (78.2)	41 (80.4)	0.77
Admission GCS	7.0 (5.0–8.0)	5.0 (3.0–8.0)	**0.010**	7.0 (5.0–8.0)	5.0 (3.0–8.5)	0.090
Number of reactive pupils on admission	2.0 (2.0–2.0)	2.0 (1.0–2.0)	0.060	2.0 (1.3–2.0)	2.0 (1.0–2.0)	0.23
Marshall CT Score	3.0 (3.0–5.0)	4.0 (2.0–5.0)	0.82	3 (2.3–5.0)	4.0 (2.3–5.0)	0.28
Subject average PRx	0.10 (0.01–0.21)	0.15 (0.03–0.29)	0.080	0.08 (0.01–0.21)	0.18 (0.04–0.29)	**0.040**
Subject average COx	0.01 (− 0.05 to 0.07)	0.03 (− 0.03 to 0.11)	0.080	0.01 (-0.05–0.05)	0.03 (− 0.02 to 0.14)	**0.010**
Subject average COx_a	0.04 (0.00–0.10)	0.05 (0.01–0.12)	0.18	0.03 (0.00–0.09)	0.07 (0.01–0.13)	**0.030**
Subject average rSO_2_	70.2 (63.2–75.9)	69.5 (64.2–74.9)	0.94	69.5 (64.3–74.7)	70.3 (62.6–76.3)	0.98

Bolded *p* values indicate statistical significance (*p* < 0.05)

*COx* Cerebral Oxygen Index Based on Cerebral Perfusion Pressure; *COx_a* Cerebral Oxygen Index Based on Arterial Blood Pressure; *CT* Computed Tomography; *GCS* Glasgow Coma Scale; *ICP* Intracranial Pressure; *IQR* Interquartile Range; *PRx* Pressure Reactivity Index; and *rSO*_*2*_ Regional Cerebral Oxygen Saturation

### Threshold value determination

**Fig. 2:**
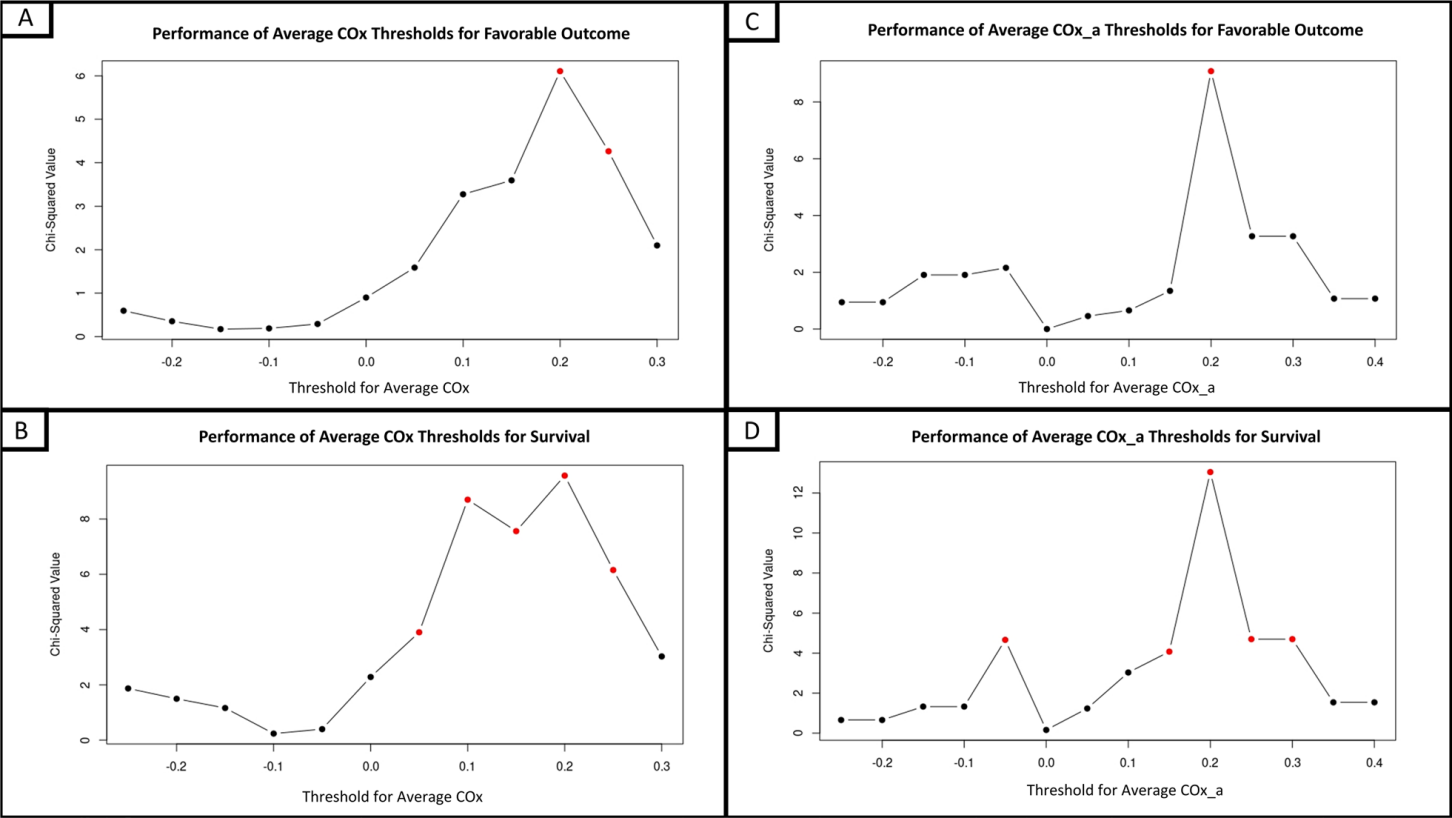
Plots of chi-square results for incremental thresholds of the cerebral perfusion pressure variant of the cerebral oxygen index (COx) and the arterial blood pressure variant of the cerebral oxygen index (COx_a). Panels **A** and **C** display the discriminative value of thresholds for favorable outcomes for average COx and COx_a, respectively. Panels **B** and **D** display the discriminative value of thresholds for survival for average COx and Cox_a, respectively. Red points indicate chi-square values that reached statistical significance (*p* < 0.05). Of note, while COx and COx_a theoretically extend from − 1 to + 1, in this dataset average values extended over a more limited range

There was no statistically significant discriminative threshold of average rSO_2_ for survival or favorable outcome. In Fig. 2A and B, + 0.2 was the threshold for average COx that produced the best statistically significant discriminative value for both favorable outcome and survival (*χ*^2^ = 6.11, *p* = 0.013; *χ*^2^ = 9.57, *p* = 0.0020, respectively). Similarly, in Fig. 2C and D + 0.2 also produced the most discriminative threshold for average COx_a for both survival and favorable outcome (*χ*^2^ = 9.08, *p* = 0.0026; *χ*^2^ = 13.04, *p* < 0.001, respectively). Full results can be seen in Additional file 1.

There was no threshold value of rSO_2_ where percentage time below was a statistically significant univariate logistic regressor for either survival or favorable outcome. Regarding COx, a threshold value of − 0.05 produced the best performing univariate logistic model with percent time over threshold as a significant regressor for survival (*p* < 0.001, AUC = 0.70, 95% CI 0.60–0.79) and for favorable outcome (*p* = 0.003, AUC = 0.65, 95% CI 0.55–0.79). Similarly, percent time over a threshold COx_a value of − 0.05 as a univariate regressor was found to produce the best performing model for survival (*p* < 0.001, AUC = 0.70, 95% CI 0.61–0.80) and favorable outcome (*p* = 0.002, AUC = 0.65, 95% CI 0.56–0.75). Full results can be seen in Additional file 2.

### Univariate and multivariable logistic regression analysis

Through the univariate logistic regression analysis, average rSO_2_ was again not found to be prognostic as a stand-alone parameter. Conversely, subject average values of ICP, PRx, COx, and COx_a were all found to be statistically significant predictors of survival and favorable outcome. Of note, average COx performed the best as a univariate regressor with the greatest degree of variance in outcome explained as demonstrated by the Nagelkerke R^2^. Generally, rSO_2_ based indices of CVR were prognostically equivalent to or better than PRx. The full results of the univariate logistic regression analysis can be found in Table 3.

**Table 3:** Univariate logistic regression

Regressor	Favorable vs unfavorable outcome				Alive vs dead			
	AUC (95% CI)	AIC	Nagelkerke R^2^	*p* value	AUC (95% CI)	AIC	Nagelkerke R^2^	*p* value
Subject average ICP	0.59 (0.49–0.69)	174.61	0.055	**0.027**	0.61 (051–0.72)	168.58	0.077	**0.010**
Subject average PRx	0.59 (0.49–0.69)	171.71	0.044	**0.047**	0.61 (0.51–0.71)	167.13	0.062	**0.019**
Subject average COx	0.59 (0.49–0.69)	172.85	0.060	**0.021**	0.63 (0.53–0.73)	163.57	0.117	**0.0016**
Subject average COx_a	0.57 (0.47–0.67)	177.00	0.044	**0.046**	0.61 (0.51–0.71)	168.32	0.089	**0.0059**
Subject average rSO_2_	0.50 (0.40–0.60)	181.13	0.002	0.66	0.50 (0.39–0.60)	176.84	0.003	0.59

Bolded *p* values indicate statistical significance (*p* < 0.05)

*AIC* Akaike Information Criterion; *AUC* Area Under the Receiver Operating Characteristic Curve; *COx* Cerebral Oxygen Index Based on Cerebral Perfusion Pressure; *COx_a* Cerebral Oxygen Index Based on Arterial Blood Pressure; *CI* Confidence Interval; *ICP* Intracranial Pressure; *PRx* Pressure Reactivity Index; and *rSO*_*2*_ Regional Cerebral Oxygen Saturation

Through the multivariable logistic regression analysis, it was found the addition of rSO_2_ to the baseline model failed to improve model performance and did not account for any additional variance in outcomes. While AUC values were similar for all models that utilized averages parameters over the recording period, Nagelkerke R^2^ values were markedly improved when the base model was augmented with average ICP, PRx, COx, or COx_a, indicating an increased ability of the model to explain outcome variance. Average ICP, COx, and COx_a remained independent predictors of survival when added to the base model, while, of these, only average ICP and average COx were independent predictors of favorable outcome. Interestingly, the multivariate models that performed the best were those that incorporated percent time over the previously determined COx and COx_a threshold of − 0.05. Of note, average PRx was neither an independent predictor of survival nor favorable outcome when base model parameters were accounted for. The full results of the multivariable logistic regression analysis can be found in Table 4.

**Table 4:** Multivariable logistic regression

Model	Favorable vs unfavorable outcome				Alive vs dead			
	AUC (95% CI)	AIC	Nagelkerke R^2^	*p* value of additional parameter	AUC (95% CI)	AIC	Nagelkerke R^2^	*p* value of additional parameter
Base model	0.73 (0.64–0.82)	164.50	0.195	N/A	0.74 (0.64–0.83)	155.64	0.231	N/A
Base model + ICP	0.75 (0.67–0.84)	158.23	0.258	**0.024**	0.78 (0.69–0.86)	144.99	0.332	**0.003**
Base Model + PRx	0.75 (0.66–0.83)	157.00	0.238	0.115	0.76 (0.67–0.85)	149.26	0.277	0.080
Base model + average COx	0.76 (0.67–0.84)	156.87	0.260	**0.038**	0.76 (068–0.85)	146.97	0.310	**0.010**
Base model + average COx_a	0.75 (0.67–0.84)	162.59	0.230	0.059	0.76 (0.67–0.85)	151.74	0.283	**0.021**
Base Model + percent Time COx > − 0.05	0.77 (0.69–0.85)	154.32	0.282	**0.010**	0.79 (0.70–0.87)	142.94	0.343	**0.002**
Base Model + percent Time COx_a > − 0.05	0.76 (0.68–0.85)	157.79	0.271	**0.006**	0.79 (0.70–0.87)	145.32	0.336	** < 0.001**
base model + rSO_2_	0.73 (0.64–0.82)	166.45	0.195	0.830	0.74 (0.65–0.83)	157.64	0.231	0.935

Bolded *p* values indicate statistical significance (*p* < 0.05)

Base model comprising of known clinical and radiographic prognostic indicators from standard prognostic models (age, admission GCS, admission pupil exam, and Marshall CT score) [35]

The base model included age, admission Glasgow Coma Scale, admission pupil reactivity, and Marshal computed tomography scale

*AIC* Akaike Information Criterion; *AUC* Area Under the Receiver Operating Characteristic Curve; *COx* Cerebral Oxygen Index Based on Cerebral Perfusion Pressure; *COx_a* Cerebral Oxygen Index Based on Arterial Blood Pressure; *CI* Confidence Interval; *ICP* Intracranial Pressure; *PRx* Pressure Reactivity Index; and *rSO*_*2*_ Regional Cerebral Oxygen Saturation

## Discussion

Through this multi-institutional retrospective cohort study, the prognostic utility of various NIRS-based parameters has been assessed. This was done using the largest multicenter database, to our knowledge, with high-frequency rSO_2_, ICP and ABP physiologic streams concurrently monitored. Given the relatively large cohort size and multi-institutional nature of the cohort, the findings of this study are likely widely generalizable to TBI populations. A key finding of this study is the prognostic utility of rSO_2_-based measures of CVR. These NIRS-based indices performed equally to the more commonly utilized ICP-based index PRx. This is in keeping with recent work indicating that PRx and COx/COx_a have a linear relationship with one another [37]. COx and COx_a, like PRx, have a range from − 1 to + 1 with higher values indicative of increased correlation of CBV and driving pressure and essentially disruption of CVR. Both COx and COx_a were found to have a grand mean threshold value of 0.2, above which outcomes worsened when the entire recording period was considered. This is consistent with previous large animal studies that found COx and COx_a values above the range of 0.3–0.5 to be associated with a complete loss of cerebral autoregulation [31, 38–40]. Given that this was the threshold found when values were averaged over the course of monitoring, a value of 0.2 may indicate a significant period of exposure to dysfunctional cerebral autoregulation resulting in poor clinical outcomes. Prior studies examining outcome associated thresholds with continuous ICP and transcranial Doppler (TCD)-based indices of CVR have also identified values in the range of 0.2 [22–24, 41–43]. These findings are also consistent with past studies in humans that have found that NIRS-based indices of CVR track strongly with ICP-based indices [15, 32, 37, 44–46].

Even just exposure to periods of disrupted CVR may be associated with worse clinical outcomes [3]. When trying to identify an exposure-based threshold, percent time over − 0.05 was found to have the best discriminative value for both COx and COx_a. This may be a more clinically useful threshold when developing CVR guided management at the bedside. While an average COx or COx_a greater than 0.2 may represent gross CVR dysfunction, an increased proportion of time above values of − 0.05 may expose the brain to deleterious effect of CVR dysfunction. It should be noted that the present study represents the first such evidence of an outcome association of these NIRS-based indices in TBI. Previous studies examining the outcome association of NIRS-based indices in TBI were likely underpowered [7].

Another notable finding of this study is that rSO_2_, as a raw NIRS parameter, failed to demonstrate any prognostic value in both univariate and multivariable analysis. Further, there was no identifiable threshold value of rSO_2_ at which outcomes following TBI worsened. This is somewhat surprising given the existing body of evidence indicating a relationship between rSO_2_ and measures of CBF or CBV [8]. A key factor to take into consideration is that in these studies it was typical that relative changes in CBV/CBF were associated with changes in rSO_2_. In the present study, absolute values of rSO_2_ were examined as a prognostic tool. This may explain why rSO_2_-based indices had more prognostic utility than raw rSO_2_ as these indices relay primary on changes in rSO_2_ mathematically as they are derived utilizing a Pearson correlation coefficient.

A key takeaway from this study is that rSO_2_ as a raw absolute parameter provides little, if any prognostic value in the ICU. This is consistent with recent studies that found high-resolution rSO_2_ signals to have differing statistical properties to more established monitoring modalities in moderate-to-severe TBI including brain tissue oxygenation (PbtO_2_), CPP, and ICP, as well as various measures of CVR [47, 48]. It should be noted that in alternative settings, such as intraoperative monitoring, change in rSO_2_ is viewed as a more reliable metric of cerebral hypoxia and ischemic risk [49, 50]. However, in the setting of moderate and severe TBI, change in rSO_2_ is a far less clear concept. Identifying an appropriate baseline in this setting is difficult given the confounders of already present cerebral injury and potentially concurrent multisystem trauma.

### Study limitations

Interpretation of the findings of this study should be taken with consideration of its limitations. First and foremost, this was a retrospective observational study where not all possible confounders were able to be accounted for. Measures of blood oxygen content such as systemic oxygen saturation (SpO_2_) and arterial partial pressure of oxygen (PaO_2_) along with other influential parameters such as end-tidal carbon dioxide (ETCO_2_) and vasoactive medication use were not available on a minute-by-minute basis. These are factors that can also modify rSO_2_ signals, and therefore rSO_2_-based indices of CVR [51]. While in the ICU setting these parameters are typically well controlled, the effect of these factors was not directly accounted for. Similarly, the degree of systemic injury or decisions surrounding withdrawal of care were not accounted for in each subject as this data was not available. This obviously neglects the effects these factors may have had on clinical outcome.

Secondly, only a single NIRS channel per patient was utilized for analysis. This was done to simplify the nature of the analysis but does mean that hemispheric differences in rSO_2_, COx, or COx_a were not examined. Additionally, the selection of which channel to use was not always able to be guided by radiographic evidence of disruptive artifacts such as extravascular blood.

Finally, analysis was conducted over the entire recording period of relevant physiologic parameters. This does mean that the temporal evolution of these parameters was not fully considered.

### Future directions

Prior to abandoning rSO_2_ as a parameter to guide management, further work will be required to examine if changes in rSO_2_ are of greater prognostic value. Consideration will need to be given to the various challenges in establishing an appropriate baseline. This will inform how best to utilize rSO_2_ at the bedside to guide care as the results of this study indicate that raw values are likely of little value. Ultimately, rSO_2_ informed management of TBI should be examined in a prospective randomized fashion to determine if this modality is a useful adjunct to more established monitoring methods.

Regarding the rSO_2_-based indices of CVR, this study has indicated that there may be significant promise in their role as a prognostic tool. This will need to be further validated in large prospective studies. Patient-specific optimal CPP targets based on COx and COx_a warrant further exploration. Whether a threshold of 0.2 or − 0.05 would produce better clinical outcomes when utilized to optimize CPP goals will need to be evaluated in a large multicenter prospective trial.

Finally, given the possible entirely noninvasive nature of COx_a, examination of the evolution of dysfunctional CVR following TBI should be possible into the chronic phase [52, 53]. A more thorough understanding of the trajectory of these parameters may help to not only guide management of this population in the acute phase but also through into the rehabilitation phase.

## Conclusion

In this study, the prognostic utility of rSO_2_ and rSO_2_-based parameters of CVR was explored in the setting of moderate-to-severe TBI. While absolute rSO_2_ as a raw parameter was found to have negligible prognostic value, COx and COx_a were both found to contain prognostic information. Beyond this, a grand mean threshold value of 0.2 was identified, for both COx and COx_a, above which outcomes markedly worsened. Additionally, − 0.05 for COx and COx_a was identified as a threshold value where the percentage of time above this threshold produced the best discriminative values. This work opens the door for further exploration of NIRS-based indices of CVR as a possible prognostic tool. Further work is needed to validate these findings and explore the possibly of COx/COx_a driven management of TBI in both the acute and chronic phases of recovery from TBI.

### Supplementary Information

**Additional file 1.** Results for grand average threshold search for rSO2, COx, and COx_a

**Additional file 2.** Results for percent time over threshold search for rSO2, COx, and COx_a

## Data Availability

The datasets analyzed and code utilized during the current study are available from the corresponding author on reasonable request.

## Abbreviations

ABP: Arterial blood pressure
AIC: Akaike information criterion
AUC: Area under the receiver operating characteristic curve
BLAS: Basic linear algebra subprograms
CAHR-TBI: CAnadian High-Resolution TBI
CBF: Cerebral blood flow
CBV: Cerebral blood volume
CI: Confidence interval
COx: Cerebral oxygen index with cerebral perfusion pressure
COx_a: Cerebral oxygen index with arterial blood pressure
CPP: Cerebral perfusion pressure
csv: Comma-separated values
CT: Computed tomography
CVR: Cerebrovascular reactivity
GCS: Glasgow Coma Scale
GOSE: Extended Glasgow Outcome Scale
ICP: Intracranial pressure
ICU: Intensive care units
IQR: Interquartile ranges
LAPACK: Linear algebra package
NIRS: Near-infrared spectroscopy
PaCO_2_: Partial pressure of arterial carbon
PaO_2_: Partial pressure of arterial oxygen
PbtO_2_: Brain tissue oxygenation
PRx: Pressure reactivity index
rSO_2_: Regional cerebral oxygen saturation
SpO_2_: Systemic oxygen saturation
TBI: Traumatic brain injury
TCD: Transcranial Doppler
