# Supplementary material for: Prognostic value of near-infrared spectroscopy regional oxygen saturation and cerebrovascular reactivity index in acute traumatic neural injury: a CAnadian High-Resolution Traumatic Brain Injury (CAHR-TBI) Cohort Study
Source: Crit Care. 2024 Mar 14;28:78. doi: 10.1186/s13054-024-04859-6 (PMC10938687; doi:10.1186/s13054-024-04859-6)
Supplement: Supplementary file 1 — Additional file 1. Results for grand average threshold search for rSO2, COx, and COx_a [file 13054_2024_4859_MOESM1_ESM.docx]

**Additional File 1**

Reported here are the various Chi Squared statistical values utilised to identify the most discriminative threshold value for grand average of rSO_2_, COx, and COx_a. Notably, no threshold reached statistical significance for rSO_2_ while 0.2 was found to be the optimal threshold value for COX and COx_a regarding survival and favorable outcome.

**Additional File 1 Table 1 – Results of Chi Squared Analysis to Identify a Grand Average rSO­_2_ Threshold for Survival and Favorable Outcome**

| **Recording Average Threshold** | **rSO_2_** | | | |
| --- | --- | --- | --- | --- |
|  | **Survival** | | **Favorable** | |
|  | **p-value** | **Chi Squared Statistic** | **p-value** | **Chi Squared Statistic** |
| **45** | 0.760308 | 0.093072 | 0.964454 | 0.001986 |
| **50** | 0.760308 | 0.093072 | 0.964454 | 0.001986 |
| **55** | 0.163908 | 1.937792 | 0.360094 | 0.837567 |
| **60** | 0.265733 | 1.238643 | 0.318678 | 0.994366 |
| **65** | 0.925612 | 0.008717 | 0.863359 | 0.029618 |
| **70** | 0.369783 | 0.804393 | 0.385017 | 0.754621 |
| **75** | 0.420098 | 0.650041 | 0.681336 | 0.168626 |
| **80** | 0.278216 | 1.175784 | 0.486471 | 0.484323 |
| **85** | 0.385289 | 0.753758 | 0.522614 | 0.408732 |
| **90** | 0.24911 | 1.328286 | 0.167118 | 1.90861 |

*rSO­_2_ = Regional Cerebral Oxygen Saturation.*

**Additional File 1 Table 2 – Results of Chi Squared Analysis to Identify a Grand Average COx Threshold for Survival and Favorable Outcome**

| **Recording Average Threshold** | **COx** | | | |
| --- | --- | --- | --- | --- |
|  | **Survival** | | **Favorable** | |
|  | **p-value** | **Chi Squared Statistic** | **p-value** | **Chi Squared Statistic** |
| **-0.25** | 0.171514 | 1.869662 | 0.440304 | 0.595491 |
| **-0.2** | 0.221391 | 1.495334 | 0.552443 | 0.352958 |
| **-0.15** | 0.281266 | 1.160962 | 0.677948 | 0.172445 |
| **-0.1** | 0.62774 | 0.235139 | 0.663206 | 0.189652 |
| **-0.05** | 0.528798 | 0.396701 | 0.589033 | 0.291857 |
| **0** | 0.130749 | 2.283593 | 0.343048 | 0.899007 |
| **0.05** | 0.048239 | 3.901638 | 0.207655 | 1.587702 |
| **0.1** | 0.003179 | 8.701644 | 0.070334 | 3.275202 |
| **0.15** | 0.005957 | 7.563261 | 0.057966 | 3.594632 |
| **0.2** | **0.001976** | **9.572069** | **0.013468** | **6.106545** |
| **0.25** | 0.013107 | 6.154631 | 0.038919 | 4.26441 |
| **0.3** | 0.081835 | 3.028078 | 0.14751 | 2.097815 |

*COx = Cerebral Perfusion Pressure Based Cerebral Oxygen Index.*

**Additional File 1 Table 3 – Results of Chi Squared Analysis to Identify a Grand Average COx_a Threshold for Survival and Favorable Outcome**

| **Recording Average Threshold** | **COx_a** | | | |
| --- | --- | --- | --- | --- |
|  | **Survival** | | **Favorable** | |
|  | **p-value** | **Chi Squared Statistic** | **p-value** | **Chi Squared Statistic** |
| **-0.25** | 0.416929 | 0.658954 | 0.440304 | 0.595491 |
| **-0.2** | 0.416929 | 0.658954 | 0.552443 | 0.352958 |
| **-0.15** | 0.24911 | 1.328286 | 0.677948 | 0.172445 |
| **-0.1** | 0.24911 | 1.328286 | 0.663206 | 0.189652 |
| **-0.05** | 0.030828 | 4.662488 | 0.589033 | 0.291857 |
| **0** | 0.687238 | 0.162092 | 0.343048 | 0.899007 |
| **0.05** | 0.266458 | 1.234892 | 0.207655 | 1.587702 |
| **0.1** | 0.08172 | 3.030356 | 0.070334 | 3.275202 |
| **0.15** | 0.043552 | 4.073849 | 0.057966 | 3.594632 |
| **0.2** | **0.000304** | **13.04424** | **0.013468** | **6.106545** |
| **0.25** | 0.030207 | 4.697479 | 0.038919 | 4.26441 |
| **0.3** | 0.030207 | 4.697479 | 0.14751 | 2.097815 |
| **0.35** | 0.214415 | 1.54136 | 0.440304 | 0.595491 |
| **0.4** | 0.214415 | 1.54136 | 0.552443 | 0.352958 |

*COx_a = Arterial Blood Pressure Based Cerebral Oxygen Index.*
