# Supplementary material for: Prognostic value of near-infrared spectroscopy regional oxygen saturation and cerebrovascular reactivity index in acute traumatic neural injury: a CAnadian High-Resolution Traumatic Brain Injury (CAHR-TBI) Cohort Study
Source: Crit Care. 2024 Mar 14;28:78. doi: 10.1186/s13054-024-04859-6 (PMC10938687; doi:10.1186/s13054-024-04859-6)
Supplement: Supplementary file 2 — Additional file 2. Results for percent time over threshold search for rSO2, COx, and COx_a [file 13054_2024_4859_MOESM2_ESM.docx]

**Additional File 2**

Reported here are the various statistical values utilised to identify the most discriminative threshold value for percent time over threshold of rSO_2_, COx, and COx_a. Notably, no threshold reached statistical significance for rSO_2_ while -0.05 was found to be the optimal threshold value for COX and COx_a regarding survival and favorable outcome.

**Additional File 2 Table 1 – Results of the Univariate Logistic Regression Modeling of Percent Time Over rSO­_2_ Threshold with Survival and Favorable Outcome**

| **Percent Time Over Threshold** | **rSO_2_** | | | | | | | |
| --- | --- | --- | --- | --- | --- | --- | --- | --- |
|  | **Survival** | | | | **Favorable** | | | |
|  | **p-value** | **AUC** | **Lower CI** | **Upper CI** | **p-value** | **AUC** | **Lower CI** | **Upper CI** |
| **30** | 0.551561 | 0.498115 | 0.405701 | 0.590529 | 0.621202 | 0.469453 | 0.37841 | 0.560495 |
| **35** | 0.614471 | 0.487431 | 0.393573 | 0.581289 | 0.672977 | 0.458089 | 0.366764 | 0.549414 |
| **40** | 0.338744 | 0.531926 | 0.436855 | 0.626997 | 0.434663 | 0.554252 | 0.462701 | 0.645804 |
| **45** | 0.280879 | 0.550654 | 0.452903 | 0.648404 | 0.416702 | 0.572214 | 0.479214 | 0.665214 |
| **50** | 0.244697 | 0.534691 | 0.432653 | 0.636728 | 0.373149 | 0.55022 | 0.453113 | 0.647327 |
| **55** | 0.368682 | 0.524384 | 0.421271 | 0.627498 | 0.537214 | 0.522239 | 0.422451 | 0.622026 |
| **60** | 0.303188 | 0.514077 | 0.408471 | 0.619684 | 0.480398 | 0.51393 | 0.41244 | 0.615419 |
| **65** | 0.703849 | 0.508798 | 0.40153 | 0.616067 | 0.67675 | 0.501711 | 0.399671 | 0.60375 |
| **70** | 0.842893 | 0.501885 | 0.39594 | 0.607831 | 0.941725 | 0.48717 | 0.385121 | 0.589219 |
| **75** | 0.682719 | 0.493841 | 0.387236 | 0.600446 | 0.802974 | 0.481061 | 0.378652 | 0.58347 |
| **80** | 0.500222 | 0.558824 | 0.457161 | 0.660487 | 0.686662 | 0.546188 | 0.446067 | 0.646308 |
| **85** | 0.391797 | 0.561337 | 0.464676 | 0.657998 | 0.661213 | 0.5435 | 0.447848 | 0.639151 |
| **90** | 0.393572 | 0.541981 | 0.453032 | 0.63093 | 0.410181 | 0.528837 | 0.440256 | 0.617417 |

*AUC = Area in the Receiver Operator Characteristic Curve; CI = Confidence Interval; rSO­_2_ = Regional Cerebral Oxygen Saturation.*

**Additional File 2 Table 2 – Results of the Univariate Logistic Regression Modeling of Percent Time Over COx Threshold with Survival and Favorable Outcome**

| **Percent Time Over Threshold** | **COx** | | | | | | | |
| --- | --- | --- | --- | --- | --- | --- | --- | --- |
|  | **Survival** | | | | **Favorable** | | | |
|  | **p-value** | **AUC** | **Lower CI** | **Upper CI** | **p-value** | **AUC** | **Lower CI** | **Upper CI** |
| **-0.95** | 0.385845 | 0.547472 | 0.46819 | 0.626754 | 0.319715 | 0.508821 | 0.42765 | 0.589991 |
| **-0.9** | 0.121185 | 0.581914 | 0.483069 | 0.680759 | 0.171961 | 0.544985 | 0.445205 | 0.644764 |
| **-0.85** | 0.050929 | 0.606166 | 0.50609 | 0.706242 | 0.068545 | 0.583543 | 0.483893 | 0.683194 |
| **-0.8** | 0.030172 | 0.634675 | 0.534794 | 0.734556 | 0.044417 | 0.611643 | 0.512594 | 0.710692 |
| **-0.75** | 0.034444 | 0.632869 | 0.531956 | 0.733782 | 0.058259 | 0.602949 | 0.503309 | 0.702588 |
| **-0.7** | 0.026119 | 0.626677 | 0.525668 | 0.727686 | 0.04717 | 0.597278 | 0.497423 | 0.697133 |
| **-0.65** | 0.014091 | 0.638029 | 0.53869 | 0.737367 | 0.034997 | 0.606603 | 0.507856 | 0.705349 |
| **-0.6** | 0.010308 | 0.644737 | 0.54478 | 0.744694 | 0.03057 | 0.607863 | 0.508646 | 0.70708 |
| **-0.55** | 0.008215 | 0.637642 | 0.537592 | 0.737692 | 0.028692 | 0.600554 | 0.50084 | 0.700269 |
| **-0.5** | 0.006403 | 0.643963 | 0.543913 | 0.744012 | 0.022846 | 0.607359 | 0.508054 | 0.706664 |
| **-0.45** | 0.005659 | 0.642931 | 0.542367 | 0.743495 | 0.02842 | 0.599546 | 0.499527 | 0.699566 |
| **-0.4** | 0.004218 | 0.646285 | 0.545361 | 0.747209 | 0.026748 | 0.602319 | 0.502271 | 0.702366 |
| **-0.35** | 0.002557 | 0.657379 | 0.556581 | 0.758177 | 0.020745 | 0.612651 | 0.513049 | 0.712254 |
| **-0.3** | 0.001439 | 0.666925 | 0.566543 | 0.767306 | 0.014132 | 0.622984 | 0.523927 | 0.722041 |
| **-0.25** | 0.000898 | 0.670021 | 0.570362 | 0.769679 | 0.01152 | 0.623992 | 0.525199 | 0.722785 |
| **-0.2** | 0.000463 | 0.678277 | 0.578715 | 0.777839 | 0.008426 | 0.628024 | 0.529412 | 0.726637 |
| **-0.15** | 0.00038 | 0.683953 | 0.584975 | 0.78293 | 0.007246 | 0.63256 | 0.534413 | 0.730708 |
| **-0.1** | 0.000213 | 0.693498 | 0.595797 | 0.7912 | 0.004361 | 0.643145 | 0.5459 | 0.74039 |
| **-0.05** | **0.000147** | **0.698658** | **0.602581** | **0.794736** | **0.002729** | **0.650706** | **0.554212** | **0.747199** |
| **0** | 0.103177 | 0.562178 | 0.4578 | 0.666555 | 0.370701 | 0.525958 | 0.424042 | 0.627874 |
| **0.05** | 0.055906 | 0.576109 | 0.472118 | 0.680101 | 0.252774 | 0.536794 | 0.435046 | 0.638543 |
| **0.1** | 0.032703 | 0.586171 | 0.482016 | 0.690327 | 0.172179 | 0.546623 | 0.444936 | 0.64831 |
| **0.15** | 0.027146 | 0.593653 | 0.489513 | 0.697794 | 0.138702 | 0.553427 | 0.451955 | 0.6549 |
| **0.2** | 0.029943 | 0.593137 | 0.488757 | 0.697518 | 0.144574 | 0.554435 | 0.453066 | 0.655805 |
| **0.25** | 0.031372 | 0.585655 | 0.480346 | 0.690964 | 0.159164 | 0.548387 | 0.44665 | 0.650124 |
| **0.3** | 0.027876 | 0.581785 | 0.475229 | 0.688341 | 0.134362 | 0.550907 | 0.449106 | 0.652709 |
| **0.35** | 0.037432 | 0.580237 | 0.473578 | 0.686896 | 0.172444 | 0.547379 | 0.445439 | 0.649319 |
| **0.4** | 0.057607 | 0.573787 | 0.467432 | 0.680142 | 0.224184 | 0.543599 | 0.441716 | 0.645482 |
| **0.45** | 0.044639 | 0.574561 | 0.468296 | 0.680827 | 0.19775 | 0.542843 | 0.4408 | 0.644885 |
| **0.5** | 0.046996 | 0.569143 | 0.463043 | 0.675244 | 0.214451 | 0.535282 | 0.433255 | 0.637309 |
| **0.55** | 0.067713 | 0.553406 | 0.446082 | 0.660729 | 0.262928 | 0.521925 | 0.419367 | 0.624484 |
| **0.6** | 0.067644 | 0.545408 | 0.438236 | 0.652579 | 0.252305 | 0.515625 | 0.413105 | 0.618145 |
| **0.65** | 0.061977 | 0.451754 | 0.34443 | 0.559079 | 0.222828 | 0.481855 | 0.379179 | 0.584531 |
| **0.7** | 0.054155 | 0.451496 | 0.344278 | 0.558715 | 0.199304 | 0.484879 | 0.382294 | 0.587464 |
| **0.75** | 0.100709 | 0.481037 | 0.373821 | 0.588253 | 0.272522 | 0.503906 | 0.401473 | 0.60634 |
| **0.8** | 0.125734 | 0.493937 | 0.387246 | 0.600628 | 0.32634 | 0.513231 | 0.411081 | 0.615381 |
| **0.85** | 0.166952 | 0.497807 | 0.391204 | 0.60441 | 0.377532 | 0.481855 | 0.37963 | 0.58408 |
| **0.9** | 0.234776 | 0.537926 | 0.431633 | 0.644219 | 0.5053 | 0.548135 | 0.446659 | 0.649611 |
| **0.95** | 0.236991 | 0.523478 | 0.424279 | 0.622677 | 0.451231 | 0.510711 | 0.413958 | 0.607463 |

*AUC = Area in the Receiver Operator Characteristic Curve; CI = Confidence Interval; COx = Cerebral Perfusion Pressure Based Cerebral Oxygen Index.*

**Additional File 2 Table 3 – Results of the Univariate Logistic Regression Modeling of Percent Time Over COx_a Threshold with Survival and Favorable Outcome**

| **Percent Time Over Threshold** | **COx_a** | | | | | | | |
| --- | --- | --- | --- | --- | --- | --- | --- | --- |
|  | **Survival** | | | | **Favorable** | | | |
|  | **p-value** | **AUC** | **Lower CI** | **Upper CI** | **p-value** | **AUC** | **Lower CI** | **Upper CI** |
| **-0.95** | 0.048479 | 0.586978 | 0.5183 | 0.655656 | 0.05003 | 0.555963 | 0.482213 | 0.629713 |
| **-0.9** | 0.010533 | 0.656863 | 0.563875 | 0.749851 | 0.016276 | 0.611926 | 0.516675 | 0.707176 |
| **-0.85** | 0.014419 | 0.6546 | 0.558612 | 0.750588 | 0.013927 | 0.6261 | 0.529922 | 0.722278 |
| **-0.8** | 0.018558 | 0.64643 | 0.548828 | 0.744033 | 0.023969 | 0.614614 | 0.517016 | 0.712212 |
| **-0.75** | 0.010116 | 0.651709 | 0.554573 | 0.748845 | 0.022345 | 0.611681 | 0.513576 | 0.709787 |
| **-0.7** | 0.007383 | 0.648064 | 0.551168 | 0.744961 | 0.025153 | 0.605572 | 0.507175 | 0.703969 |
| **-0.65** | 0.004478 | 0.663147 | 0.567247 | 0.759048 | 0.019426 | 0.620479 | 0.523348 | 0.71761 |
| **-0.6** | 0.002738 | 0.673203 | 0.577935 | 0.76847 | 0.016904 | 0.629277 | 0.53271 | 0.725843 |
| **-0.55** | 0.001686 | 0.679236 | 0.584711 | 0.773761 | 0.012545 | 0.634897 | 0.538694 | 0.731101 |
| **-0.5** | 0.001952 | 0.677476 | 0.581687 | 0.773266 | 0.013519 | 0.633675 | 0.537093 | 0.730258 |
| **-0.45** | 0.001248 | 0.683007 | 0.587432 | 0.778581 | 0.00957 | 0.639541 | 0.543206 | 0.735875 |
| **-0.4** | 0.000887 | 0.687029 | 0.5907 | 0.783358 | 0.008383 | 0.641984 | 0.545521 | 0.738448 |
| **-0.35** | 0.0006 | 0.690548 | 0.593581 | 0.787515 | 0.00745 | 0.641496 | 0.544611 | 0.73838 |
| **-0.3** | 0.00036 | 0.695827 | 0.598744 | 0.792911 | 0.004523 | 0.646628 | 0.549745 | 0.74351 |
| **-0.25** | 0.000193 | 0.699598 | 0.602861 | 0.796334 | 0.003717 | 0.645406 | 0.54832 | 0.742491 |
| **-0.2** | 0.000169 | 0.697587 | 0.600811 | 0.794362 | 0.003478 | 0.641496 | 0.544641 | 0.73835 |
| **-0.15** | 0.000129 | 0.699095 | 0.602608 | 0.795582 | 0.002657 | 0.646383 | 0.550057 | 0.74271 |
| **-0.1** | 0.000137 | 0.702112 | 0.605795 | 0.798428 | 0.002563 | 0.651515 | 0.55541 | 0.74762 |
| **-0.05** | **0.000136** | **0.702866** | **0.607716** | **0.798016** | **0.002089** | **0.655547** | **0.560305** | **0.75079** |
| **0** | 0.447964 | 0.516088 | 0.409772 | 0.622405 | 0.78739 | 0.510997 | 0.409269 | 0.612725 |
| **0.05** | 0.341442 | 0.472097 | 0.365571 | 0.578622 | 0.674629 | 0.498289 | 0.396483 | 0.600095 |
| **0.1** | 0.274846 | 0.469331 | 0.362838 | 0.575824 | 0.597183 | 0.497312 | 0.395577 | 0.599047 |
| **0.15** | 0.21664 | 0.540724 | 0.434523 | 0.646925 | 0.545037 | 0.50782 | 0.406264 | 0.609376 |
| **0.2** | 0.185116 | 0.546003 | 0.440232 | 0.651774 | 0.493892 | 0.510997 | 0.40957 | 0.612424 |
| **0.25** | 0.176755 | 0.542735 | 0.436705 | 0.648765 | 0.489012 | 0.507576 | 0.406032 | 0.609119 |
| **0.3** | 0.141408 | 0.545752 | 0.43952 | 0.651983 | 0.427202 | 0.509286 | 0.407691 | 0.610882 |
| **0.35** | 0.126048 | 0.54726 | 0.441209 | 0.653311 | 0.384101 | 0.513196 | 0.411693 | 0.6147 |
| **0.4** | 0.103917 | 0.54726 | 0.441172 | 0.653348 | 0.336748 | 0.514418 | 0.412905 | 0.615932 |
| **0.45** | 0.085273 | 0.549774 | 0.443507 | 0.65604 | 0.283256 | 0.521505 | 0.420037 | 0.622974 |
| **0.5** | 0.076091 | 0.546506 | 0.440346 | 0.652666 | 0.262994 | 0.517351 | 0.415852 | 0.61885 |
| **0.55** | 0.076624 | 0.551533 | 0.445306 | 0.657761 | 0.257765 | 0.518817 | 0.417255 | 0.620379 |
| **0.6** | 0.066162 | 0.554299 | 0.448206 | 0.660392 | 0.222768 | 0.52175 | 0.420213 | 0.623286 |
| **0.65** | 0.077563 | 0.546506 | 0.440389 | 0.652622 | 0.233118 | 0.518817 | 0.417428 | 0.620206 |
| **0.7** | 0.093017 | 0.547511 | 0.441743 | 0.65328 | 0.264819 | 0.515396 | 0.413963 | 0.616829 |
| **0.75** | 0.127481 | 0.531423 | 0.425694 | 0.637152 | 0.322427 | 0.498534 | 0.397044 | 0.600023 |
| **0.8** | 0.188597 | 0.520362 | 0.415934 | 0.62479 | 0.429964 | 0.486559 | 0.385086 | 0.588032 |
| **0.85** | 0.199173 | 0.519985 | 0.415983 | 0.623987 | 0.443845 | 0.485337 | 0.383922 | 0.586752 |
| **0.9** | 0.219843 | 0.497235 | 0.393458 | 0.601011 | 0.517029 | 0.561828 | 0.46108 | 0.662576 |
| **0.95** | 0.381575 | 0.499497 | 0.400911 | 0.598083 | 0.723341 | 0.529814 | 0.433076 | 0.626553 |

*AUC = Area in the Receiver Operator Characteristic Curve; CI = Confidence Interval; COx_a = Arterial Blood Pressure Based Cerebral Oxygen Index.*
